# How personalised medicine will transform healthcare by 2030: the ICPerMed vision

**DOI:** 10.1186/s12967-020-02316-w

**Published:** 2020-04-28

**Authors:** Astrid M. Vicente, Wolfgang Ballensiefen, Jan-Ingvar Jönsson

**Affiliations:** 1 Department of Health Promotion and NCD Prevention, Instituto Nacional de Saúde Doutor Ricardo Jorge, Av Padre Cruz, 1691-016 Lisbon, Portugal; 2 Biosystems and Integrative Sciences Institute (BioISI), Faculdade de Ciências da Universidade de Lisboa, Campo Grande, Lisbon, Portugal; 3 German Aerospace Center (DLR), Project Management Agency, Innovation for disease Related Research, Health, Personalised Medicine, Heinrich-Konen-Str. 1, 53227 Bonn, Germany; 4 Swedish Research Council, Scientific Council for Medicine and Health, Box 1035, 101 38 Stockholm, Sweden; 5 Division of Molecular Medicine and Virology, Department of Biomedical and Clinical Sciences, Linköping University, 581 83 Linköping, Sweden

**Keywords:** Personalised medicine, ICPerMed, Vision for 2030, Healthcare

## Abstract

This commentary presents the vision of the International Consortium for Personalised Medicine (ICPerMed) on how personalised medicine (PM) will lead to the next generation of healthcare by 2030. This vision focuses on five perspectives: individual and public engagement, involvement of health professionals, implementation within healthcare systems, health-related data, and the development of sustainable economic models that allow improved therapy, diagnostic and preventive approaches as new healthcare concepts for the benefit of the public. We further identify four pillars representing transversal issues that are crucial for the successful implementation of PM in all perspectives. The implementation of PM will result in more efficient and equitable healthcare, access to modern healthcare methods, and improved control by individuals of their own health data, as well as economic development in the health sector.

Personalised medicine (PM) represents an exciting opportunity to improve the future of individualised healthcare for all citizens (*citizen* herein equivalent to individuals in the society, reflecting the inclusive and fair nature of PM approaches), holding much promise for disease treatment and prevention. There are high expectations for the future, but will PM and its accompanying tools and approaches change healthcare and be widely implemented for the benefit of society and its citizens by 2030? Will scientists, innovators, healthcare providers, and others be able to provide the most suitable medicine, at the right dose, for the right person, at the right time, at a reasonable cost? Will the healthcare sector be able to find the incentives and create appropriate financial models to implement PM in daily clinical practice? These are questions that require immediate attention and coordinated action to achieve the goal of the comprehensive implementation of PM already by 2030.

The International Consortium for Personalised Medicine (ICPerMed) [1] believes that advancement of the biomedical, social, and economic sciences, together with technological development, is the driving force for PM. Strong investment in research and innovation is therefore a prerequisite for its successful implementation. Here, we present our vision of how PM will lead to the next generation of healthcare by 2030. Through five main perspectives, our vision affirms PM as a medical practice centred on the individual’s characteristics, leading to improved effectiveness of diagnostics, treatment and prevention, added economic value, and equitable access for all citizens.

ICPerMed envisages healthcare within the five core perspectives, further delineated in our white paper [2], to be implemented by 2030 as follows:

Perspective 1: Informed, empowered, engaged, and responsible citizens

- Health-related data is controlled by the citizen, including input, monitoring, and access.
- Easily accessible, reliable, and understandable sources of medical information are available.

Perspective 2: Informed, empowered, engaged, and responsible health providers

- The safe, responsible, and optimal use of health information and research results required for PM is routine in clinical settings.
- Clinical decisions requiremultidisciplinary teams, integrating novel health-related professions.
- The education of healthcare professionals has adopted the interdisciplinary aspects of PM.
- Clinicians and researchers work closely to support the rapid development and implementation of PM solutions.

Perspective 3: Healthcare systems enable personally tailored, optimised health promotion and disease prevention, diagnosis, and treatment for the benefit of patients

- Equitable access to PM services for all citizens is a reality.
- PM services are optimised in terms of effectiveness and equity.
- The allocation of resources within healthcare systems is consistent with societal values.
- Secure health data flow from citizens and healthcare systems to regulatory authorities and research is in place.

Perspective 4: Available health-related information for optimised treatment, care, prevention, and research

- Personal data in electronic health records (EHRs) is used by healthcare providers and researchers for more efficient PM.
- Harmonised solutions to ensure data privacy, safety, and security are applied in health-data management.
- Optimised treatment and prevention based on personal data benefit citizens, while minimising costs and risks.

Perspective 5: Economic value by establishing the next generation of medicine

- A reasonable balance between investment, profit, and shared benefit for the citizen is a reality for PM.
- Appropriate business concepts and models are in place for PM.
- Telemedicine and mobile solutions promote PM and are of economic value.
- New jobs in healthcare systems are created.

The ICPerMed vision for 2030 is aligned with the goals of the United Nations 2030 Agenda for Sustainable Development, which sets out a vision for good health and well-being, promoting *healthy lifestyles, preventive measures, and modern, efficient healthcare for everyone*. To support these goals and sustain the five perspectives of the ICPerMed vision, four pillars representing transversal issues are crucial for the successful implementation of PM (Fig. 1):

**Fig. 1:**
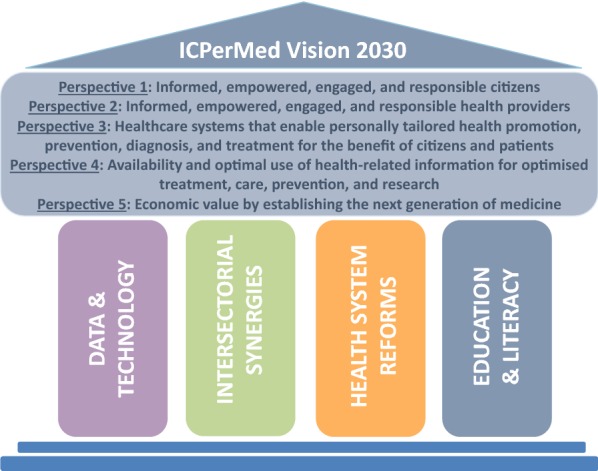
The ICPerMed vision for 2030–a framework of five perspectives addressing the role of the individual, the role of health professionals, the implementation of PM in health systems, the use of personal health-related information, and the economic value of PM approaches, sustained by four critical pillars representing transversal issues crucial for the implementation of PM

- Data and technology.

By 2030, digital technology is a ubiquitous enabler of all aspects of society, including the health and well-being of citizens. Attitudes towards digital technology and data sharing have changed, driven by a new generation for whom digital technology and social networks are fully integrated in daily life. These citizens are more empowered to control their health data than those of previous generations, and thus more engaged in healthcare decisions and data sharing for research. Adequate regulatory frameworks and data management protocols for the protection of personal rights are compliant with international state-of-the-art standards addressing data security, accessibility, storage, and curation.

Comprehensive personal health data is, in 2030, available through EHRs. The widespread use of wearable devices and apps allows continuous and real-time tracking of health parameters and behaviours, which is complemented by biomarker technology. The global efforts to understand genomic variation in millions of individuals allow the definition of individual genomic risk profiles associated with common diseases, placing greater emphasis on prevention. Other levels of biological information, including epigenomics, proteomics, and metabolomics complement genomic-risk estimates and provide monitoring tools for individuals at risk for disease. Data generation is continuously evolving, requiring innovative and flexible information and communication technology (ICT) solutions to address the needs of PM models for data storage, management, access, safety, and sharing. Interoperability and harmonisation concepts are embedded in healthcare and research systems through more homogeneous data collection tools. Significant investments in artificial intelligence methods by 2030 lead to novel and efficient integration and interpretation of multilevel data from a wide range of sources. Finally, creative and trustworthy ICT solutions are available to support clinical decisions by healthcare providers at the point-of-care.

- Inter-sectoral synergies.

In the ICPerMed vision for 2030, strong synergies between healthcare and research are crucial for the application of PM. Large volumes of routine healthcare data provide a rich source of material for research, allowing patient stratification and the definition of individual profiles and supporting adapted clinical trials. A close alignment between healthcare providers, researchers, and patients, together with improved flexibility of healthcare systems, enables end user-driven biomedical and clinical research and supports the rapid assimilation of research results by the clinic. The healthcare systems of 2030 support research to strengthen the evidence base of novel PM strategies, effectiveness, and value.

Other parameters influencing health outcomes, including lifestyle and behaviour, socio-economic status, employment, and environmental exposure are integrated with personal health and biomarker data. Acknowledging the impact of policies from other sectors enables valuable inter-sectoral synergies, particularly for health promotion and disease prevention.

In 2030, synergies with the private sector are driven by the need for rapid technological progress, along with novel business opportunities and models. PM drives innovation, particularly in areas such as digital technology, biomarker detection, and the development of molecular-targeted drugs. Through close cooperation with the pharma industry, data from clinical trials is available to the medical community, improving patient access to innovative medicines. Health technology assessment clarifies the true value of technologies, incentivizing PM.

- Healthcare system reforms.

By 2030, the primary focus of healthcare has shifted from treatment to risk definition, patient stratification, and personalised health promotion and disease prevention strategies of particular value for ageing societies. Optimisation of healthcare systems until 2030 reflects this change. Economic sustainability and societal benefits of PM are clear and integrate a societal perspective. Economic analysis is at a systemic level, integrating unemployment, social-care systems, new risk-sharing methods, and the entire life cycle of PM approaches. This broader societal perspective is underpinned by shared ethical values and equity of access for all, including marginalised sectors and under-served populations. In 2030, adequate reimbursement models are in place to support this more equitable approach, and consider the long-term value of innovative technology-based approaches.

Significant investments in technological infrastructure and digital platforms until 2030 maximize the enormous economic value of public ownership of data and create the need for new skills and novel professional profiles. Health professionals trained in digital technologies, biomarker examination, and data analysis are members of multidisciplinary teams that make shared clinical decisions. Healthcare systems use flexible working models to accommodate individual needs and incorporate the rapid turnover of technological and scientific innovations streaming from research, and bidirectional data accessibility is facilitated by networking and data-sharing platforms.

- Education and literacy.

Major changes in medical and other healthcare provider *curricula* (e.g. pharmacists, nurses, and therapists) result in a new generation of informed, empowered, engaged, and responsible healthcare providers by 2030. There is a strong focus on digital literacy and the skills needed to interpret biomarker information. The value of multidisciplinarity in clinical and healthcare decisions is routinely used in practice. Given the fast turnover of technologies and their potential impact on healthcare, lifelong education and training is essential for healthcare providers. Conversely, professionals with non-clinical backgrounds have a better understanding of healthcare and clinical issues, facilitating interactions amongst clinical teams.

For the citizen, health data education and literacy in PM, including ethical, regulatory, and data-control issues, is provided through schools and specific literacy programs. Improved PM literacy is complemented by interfaces capable of providing required rigorous information on demand while preserving the patient-clinician interaction.

In 2030, healthcare managers and policy makers have ample evidence of the benefits of PM to citizens and healthcare systems. This enables the establishment of political frameworks to tackle effectiveness, efficiency, equity, and ethical issues underlying the development and implementation of PM approaches.

## Conclusions

PM is not so much a paradigm change as the evolution of medicine in a biotechnology and data-rich era. This development requires extensive adjustments in the way healthcare is provided, including new skills for healthcare professionals and novel tools for delivery. The ICPerMed vision reflects such an evolution. It was supported by consulting European and international experts, covering key sectors, who provided feedback on the opportunities and challenges of PM and highlighted specific concerns and possible solutions [2].

ICPerMed supports coordinated research directed towards the progressive implementation of PM and has previously developed an Action Plan [3], defining research activities to stimulate the adoption of PM in healthcare. Leveraging the Action Plan, ICPerMed members have been successful in establishing PM research and healthcare programs and actions in their own countries and regions [4]. The European Commission already supports many initiatives consistent with the presented vision and, together with ICPerMed, is committed to expanding its efforts globally. The core perspectives of the ICPerMed vision and transversal issues presented herein can further orient policy makers and guide the healthcare community in their planning of future programs and activities for PM implementation. ICPerMed will continue to act as a communication platform for existing and future initiatives and organisations related to PM, paving the way towards this vision of PM in 2030.

## Data Availability

Data sharing is not applicable to this article as no datasets were generated or analysed for this commentary. The analysis of data from the mentioned survey to European and international experts is published separately [2].

## Abbreviations

PM: Personalised medicine
ICPerMed: International consortium for personalised medicine
EHR: Electronic health records
ICT: Information and communication technology

## References

[1] https://www.icpermed.eu.

[2] https://www.icpermed.eu/en/activities-vision-paper.php.

[3] Venne Julien, Busshoff Ulrike, Poschadel Sebastian, Menschel Robin, Evangelatos Nikolaos, Vysyaraju Kranthi, Brand Angela (2019). International consortium for personalised medicine: an International survey about the future of personalised medicine. Pers Med..

[4] https://www.icpermed.eu/en/activities-action-plan.php.

